# Digital Dentistry: New Materials and Techniques

**DOI:** 10.1155/2016/5261247

**Published:** 2016-10-20

**Authors:** Francesco Mangano, Jamil A. Shibli, Thomas Fortin

**Affiliations:** ^1^Department of Surgical and Morphological Science, Dental School, University of Varese, Varese, Italy; ^2^Academic Unit of Digital Dentistry, IRCCS San Raffaele Hospital, Milan, Italy; ^3^Guarulhos University, Guarulhos, SP, Brazil; ^4^University of Lyon, Lyon, France

The digital revolution is changing the world, and dentistry is no exception.

The introduction of a whole range of digital devices (intraoral, extraoral, face scanners and cone beam computed tomography (CBCT) with low dose radiation) and processing software (computer-assisted-design/computer-assisted-manufacturing (CAD/CAM) prosthetic software, software for planning implant surgery), together with new aesthetic materials and powerful manufacturing and prototyping tools (milling machines and 3D printers), is radically transforming the dental profession.

Classically, case history and physical examination, together with X-ray data from two-dimensional radiology (periapical, panoramic, and cephalometric radiographs), represented the necessary preparatory stages for formulating a treatment plan and for carrying out the therapy. With only two-dimensional X-ray data available, making a correct diagnosis and an appropriate treatment plan could be difficult; therapies essentially depended on the manual skills and experience of the operator.

Today, the digital revolution is changing the workflow and consequently changing operating procedures. In modern digital dentistry, the four basic phases of work are image acquisition, data preparation/processing, the production, and the clinical application on patients.

Image acquisition is the first operational phase of digital dentistry and this employs powerful tools such as digital cameras, intraoral scanners, and CBCT.

Digital photography, combined with the use of appropriate software for image processing, allows us to design a patient's smile virtually: this is digital smile design, a valuable tool for previsualization and communication in modern aesthetic and cosmetic dentistry.

Intraoral scanners allow us to take accurate optical impression of the dental arches, using only a beam of light. The optical impression is now supplanting the classic method with tray and impression materials: this last procedure, never liked by patients and often technically difficult, is likely to disappear over the next few years. The information on dentogingival tissues acquired from an optical impression can be used not only to make a diagnosis and for communication, but also to design prosthetic restorations. Indeed, optical impression data (e.g., the scanning of prosthetic preparations) is easily imported into processing software for designing/planning prosthetic restorations; the models created in this way are then physically produced with materials of high aesthetic value, with powerful milling machines. These restorations are then delivered to patients. In simple cases (e.g., in the case of inlays/onlays, temporary restorations in resin or single crowns), all these procedures can be performed directly in the dental office, without the help of a dental technician, through a “full in-office” or “chairside” procedure. In the case of more complex restorations (such as crowns and monolithic bridges or copings and frameworks which need to be veneered with ceramic), collaboration with a dental technician is fundamental. Optical impressions can find application in orthodontics and surgery, too. In orthodontics, they are useful in diagnosis and in the design of a whole series of custom devices, such as invisible aligners. In surgery, optical impressions allow useful data to be obtained for the planning of implant operations. Indeed, information on dentogingival tissues can be combined and overlaid with that relating to the patient's bone structure obtained from CBCT, by using specific planning software packages. Within these processing software packages, the surgeon can design templates for guided implant placement, which are physically manufactured by milling or 3D printing and used clinically. Implants positioned through a guided surgical procedure can be loaded immediately, using prosthetic restorations in resin, printed in 3D before the fixtures are positioned. This is known as the “full-digital” technique.

The data obtained by CBCT can also be used for designing personalized implants (“custom-made”), or personalized bone grafts, to be used in implant and regenerative surgery, respectively. In fact, by importing data from CBCT into specific modelling software, it is currently possible for the surgeon to design a whole series of customized implants (root analogue implants, blade implants, and maxillofacial implants); such implants can be physically produced by means of additive manufacturing and 3D printing procedures, such as direct metal laser forming (printing of metals). The use of customized implants offers the patient several advantages. With 3D printing techniques (the real “third industrial revolution”) becoming established in the biomedical field, the cost of equipment will fall and the printers will also increasingly become accessible to dental professionals: hence, it is likely that such modelling procedures and the fabrication of “customized” implants will spread. The production of personalized bone grafts for regenerative surgery also comes under this heading. The possibility of using custom-made bone grafts, offering macro- and microtopography with controlled characteristics, represents an undoubted advantage for the practitioner and for the patient. In fact, the availability of customized grafts which fit the individual patient's bone defect perfectly will greatly simplify and speed up otherwise complex regenerative operations; reducing the surgical time will allow the risk of infection to be lowered and improve healing. Thus, the outcome of regenerative therapy will also be improved with significant benefit for the patient.

In this special issue, the first in the world dedicated entirely to the topic of digital dentistry, you will find that we have gathered together a number of scientific and clinical papers which deal with various “digital” themes: we hope that you will find them interesting and that they will attract your attention.

*Francesco Mangano* *Francesco Mangano* *Jamil A. Shibli* *Jamil A. Shibli* *Thomas Fortin* *Thomas Fortin*

